# Role of the C-reactive Protein-to-Albumin Ratio in Assessing Disease Activity in Elderly Patients With Rheumatoid Arthritis

**DOI:** 10.7759/cureus.66929

**Published:** 2024-08-15

**Authors:** Sadettin Uslu, Filiz Cemre Tasgöz

**Affiliations:** 1 Rheumatology, Manisa Celal Bayar University School of Medicine, Manisa, TUR

**Keywords:** crp-to-albumin ratio (car), neutrophil-to-lymphocyte ratio (nlr), disease activity score (das28-esr), elderly population, rheumatoid arthritis

## Abstract

Objective: Nowadays, one measure that is more helpful in assessing the level of inflammation than either C-reactive protein (CRP) or albumin alone is the C-reactive protein-to-albumin ratio (CAR). Our study set out to assess the CAR in elderly individuals with rheumatoid arthritis (RA) and its correlation with other parameters.

Methods: Included in the research were patients who were being followed up on for RA between January 2021 and January 2024 and categorized according to their age at the time of enrolment and assigned to one of two groups: younger patients, defined as <60 years of age, and those aged ≥60 years, who were recorded as elderly patients. The clinical evaluation of the patients and laboratory data measured for each patient included age, gender, disease duration, medications, CRP, erythrocyte sedimentation rate (ESR), albumin, neutrophil-to-lymphocyte ratio (NLR), and CAR. Disease activity was assessed with the disease activity score 28 (DAS 28)-ESR. The health assessment questionnaire was used to measure the functional status.

Results: Ninety-four patients (<60 years: 58 and ≥60 years: 36) were included. The mean age of the elderly patients was 65.80 ± 5.33 years. Female predominance was similar in both the RA groups (<60 years: 50 patients (86.2%) vs. ≥60 years: 31 (86.1%)). The distribution of biological and disease-modifying drugs did not significantly differ between the groups. With the exception of albumin, there was no statistically significant difference between the groups for ESR, CRP, CAR, NLR, or DAS28-ESR. Elderly patients with a DAS28-ESR of 2.6 and above had a statistically significant higher CAR than the remission group (3.44±3.73 vs. 2.71±5.73, respectively). There was no statistically significant difference in the NLR value of elderly patients with a DAS28-ESR of 2.6 and above compared to the remission group (3.06 ± 2.95 vs. 2.65 ± 1.38, respectively). In addition, CAR was positively correlated with ESR, CRP, and DAS28-ESR (r = 0.726, p < 0.001; r = 0.954, p < 0.001; r = 0.339, p = 0.043, respectively). However, there was no discernible correlation between CAR and HAQ, NLR, or disease duration.

Conclusion: In elderly RA patients, our study demonstrated the correlation between CAR and inflammatory biomarkers and the DAS28-ESR. According to this, CAR may prove to be a useful biomarker for assessing inflammation and disease activity in clinical settings.

## Introduction

The elderly population is growing both in Turkey and globally. The World Health Organization (WHO) defines the elderly as those aged 60 and over. Based on these distinction criteria, it is estimated that 13% of the current world population is elderly [1]. The incidence of rheumatological disorders is rising in the elderly population at the same time that life expectancy is rising.

One of the most prevalent inflammatory autoimmune diseases is rheumatoid arthritis (RA), which is characterized by progressive joint deterioration and symmetrical polyarthritis. RA affects 0.5-1% of the adult population. Compared to men, women are three times more likely to be affected [2]. The condition is commonly diagnosed as elderly RA in people over 60, but it typically affects those between the ages of 30 and 50 [3,4].

C-reactive protein (CRP) levels are often used in addition to joint swelling and tenderness to assess disease activity. It is a part of the core set for quantifying clinical response in RA clinical trials, which is maintained by the American College of Rheumatology (ACR) [5]. Albumin levels also change in inflammatory diseases. A more accurate measure of the state of inflammation than either CRP or albumin alone is the C-reactive protein-to-albumin ratio (CAR), which is determined by dividing the CRP level by the albumin level. Recent studies have shown that the CAR, a new inflammation-based score, is associated with the inflammatory status in RA [5-7].

To our knowledge, no prior research has looked at the connection between clinical parameters and CAR in elderly RA patients. Thus, the objective of this research was to assess the correlation between CAR and RA parameters in an elderly patient population.

## Materials and methods

Patient selection

In this research, a total of 94 RA patients who presented to the rheumatology outpatient clinic at Manisa Celal Bayar University Hafsa Sultan Hospital, Manisa, Turkey, with regular controls from January 2021 to January 2024 were included. The criteria for inclusion in the study were being over 18 years of age, satisfying the 2010 RA categorization criteria established by the ACR and the European League Against Rheumatism [8], and giving informed consent to participate. Pregnancy, breastfeeding, cancer, other chronic inflammatory diseases, those who are obese, malignant hematological disorders, active hepatitis, and chronic liver or kidney failure were among the exclusion criteria. Patients have not visited an infectious disease clinic in the last two weeks. Patients were categorized by age: patients aged <60 years were classified as young, and patients aged ≥60 years were classified as elderly.

Data

Each patient's clinical and laboratory data comprised age, gender, disease duration, medications, CRP, erythrocyte sedimentation rate (ESR), albumin, neutrophil-to-lymphocyte ratio (NLR), CAR, rheumatoid factor (RF) and anti-cyclic citrullinated peptide (anti-CCP). Disease activity was assessed with the disease activity score 28 (DAS 28)-ESR. The visual analog scale, ESR measurements, and the number of swollen or painful joints were used to create the DAS28-ESR. More than 5.1 on the DAS28-ESR denotes severe disease activity, 2.6 to 5.1 on the DAS28-ESR denotes low to moderate activity, and <2.6 denotes remission [9]. Patients with a score below 2.6 constituted group 1 (patients in remission), and patients with a score of 2.6 and above constituted group 2 (patients with active disease). The Health Assessment Questionnaire (HAQ) was used to measure the functional status [10].

Ethics

Ethical approval was obtained from the Manisa Celal Bayar University Faculty of Medicine, Health Sciences Ethics Committee (Decision Date/No.: 2024/2527) before the study began. Signed informed consent was obtained from each participant to ensure adherence to ethical guidelines and respect for the rights and welfare of research participants.

Statistical analysis

Statistical analysis was performed with Statistical Package for the Social Sciences (SPSS) software, version 22.0 (IBM Corp., Armonk, NY, USA). Levene's test was used to assess homogeneity of variance, and the Shapiro-Wilk test was used to determine if the data conformed to a normal distribution. The independent-sample T-test with bootstrap findings or the Mann-Whitney U test with Monte Carlo results were employed in the quantitative variable comparison. A comparison of categorical variables was conducted using the Pearson chi-square test. Categorical variables were reported as n (%) and quantitative data as mean±SD (standard deviation). To evaluate the relationships between CAR and inflammatory markers and disease activity, Spearman's correlation analysis was employed. Utilizing receiver operating characteristic (ROC) curve analysis, the CAR parameter usefulness in determining disease activity was evaluated. Each variable was analyzed at a 95% confidence level, and a significant p-value was defined as less than 0.05.

## Results

Among a total of 94 RA patients, the mean age of the elderly patients was 65.80 ± 5.33 years and the mean age of the young patients was 48.48 ± 7.10 years (Table 1). Of the total patients, 66.0% had positive results for RF and 68.1% for anti-CCP. The presence of autoantibodies was not statistically different between the two groups. The elderly patients included 31 females (86.1%), while the young group contained 50 females (86.2%). Female predominance was similar in both RA groups (<60 years: 50 patients (86.2%) vs. ≥60 years: 31 (86.1%), p = 0.990). The distribution of biological usage and disease-modifying drugs did not significantly differ between the groups. There was no statistically significant difference between the groups in terms of ESR, CRP, CAR, NLR, and DAS28-ESR except albumin (p = 0.020) (Table 1).

**Table 1:** Demographic and clinical characteristics of the study group CRP: C-reactive protein; ESR: erythrocyte sedimentation rate; CAR: C-reactive protein to albumin ratio; NLR: neutrophil-lymphocyte ratio; HAQ: Health Assessment Questionnaire; cDMARD: conventional disease-modifying antirheumatic drug; bDMARD: biological disease-modifying antirheumatic drug; DAS: disease activity score; ANA: anti-nucleolar antibody; Rf: rheumatoid factor; CCP: cyclic citrullinated peptide. *: statistically significant at p < 0.001, **: statistically significant at p ≤ 0.05.

	Total (n = 94)	<60 (n = 58)	≥60 (n = 36)
Age (years), mean±SD	55.11±10.64	48.48±7.10	65.80±5.33*
Female, n (%)	81 (86.2)	50 (86.2)	31 (86.1)
Disease duration (month), mean±SD	61.87±83.59	54.20±82.01	74.22±85.79
Rf positive, n (%)	62 (66.0)	38 (65.5)	24 (66.7)
Anti-CCP positive, n (%)	64 (68.1)	38 (65.5)	26 (72.2)
ANA positive, n (%)	15 (16.0)	10 (17.2)	5 (13.9)
ESR (mm/h), mean±SD	39.21±23.99	39.03±24.91	39.77±22.78
CRP (mg/L), mean±SD	12.91±18.42	13.45±18.58	12.03±18.40
Albumin (g/dL), mean±SD	4.21±0.35	4.28±0.29	4.08±0.40**
DAS28-CRP, mean±SD	3.18±1.06	3.20±1.08	3.14±1.04
Moderate-high disease activity, n (%)	46 (48.9)	29 (50)	17 (47.2)
HAQ, mean±SD	0.45±0.56	0.41±0.57	0.50±0.56
CAR, mean±SD	3.17±4.55	3.24±4.51	3.05±4.67
NLR, mean±SD	2.61±1.96	2.46±1.78	2.85±2.24
Treatment			
cDMARD , n (%)	74 (78.7)	46 (79.3)	28 (77.8)
bDMARD, n (%)	16 (17.0)	11 (19.0)	5 (13.9)
Corticosteroid, n (%)	44 (46.8)	28 (48.3)	16 (44.4)

Elderly patients with a DAS 28-ESR of 2.6 and above had a statistically significant higher CAR than the remission group (3.44 ± 3.73 vs. 2.71 ± 5.73, p = 0.044). There was no statistically significant difference in the NLR value of elderly patients with a DAS 28-ESR of 2.6 and above compared to the remission group (3.06 ± 2.95 vs. 2.65 ± 1.38, respectively, p = 0.851) (Table 2). CAR was positively correlated with ESR, CRP, and DAS28-ESR (r = 0.726, p < 0.001; r = 0.954, p < 0.001 and r = 0.339, p = 0.043, respectively). However, there was no discernible correlation between CAR and HAQ, NLR, or disease duration (Table 3).

**Table 2:** Comparison of CAR and NLR according to disease activation in elderly RA patients CAR: C-reactive protein-to-albumin ratio; NLR: neutrophil-to-lymphocyte ratio. *: statistically significant at p ≤ 0.05.

	Remission (n = 19)	Moderate-high (n = 17)
CAR, mean±SD	2.71±5.73	3.44±3.73*
NLR, mean±SD	2.65±1.38	3.06±2.95

**Table 3:** Correlation of C-reactive protein/albumin ratio with other clinical parameters CRP: C-reactive protein; ESR: erythrocyte sedimentation rate; CAR: C-reactive protein-to-albumin ratio; NLR: neutrophil-to-lymphocyte ratio; HAQ: Health Assessment Questionnaire; DAS: disease activity score. *: statistically significant at p < 0.001, **: statistically significant at p ≤ 0.05.

	R
ESR (mm/h)	0.726*
CRP (mg/L)	0.954*
DAS28-ESR	0.339**
Disease duration	0.123
HAQ	0.121
NLR	0.264

**Figure 1:**
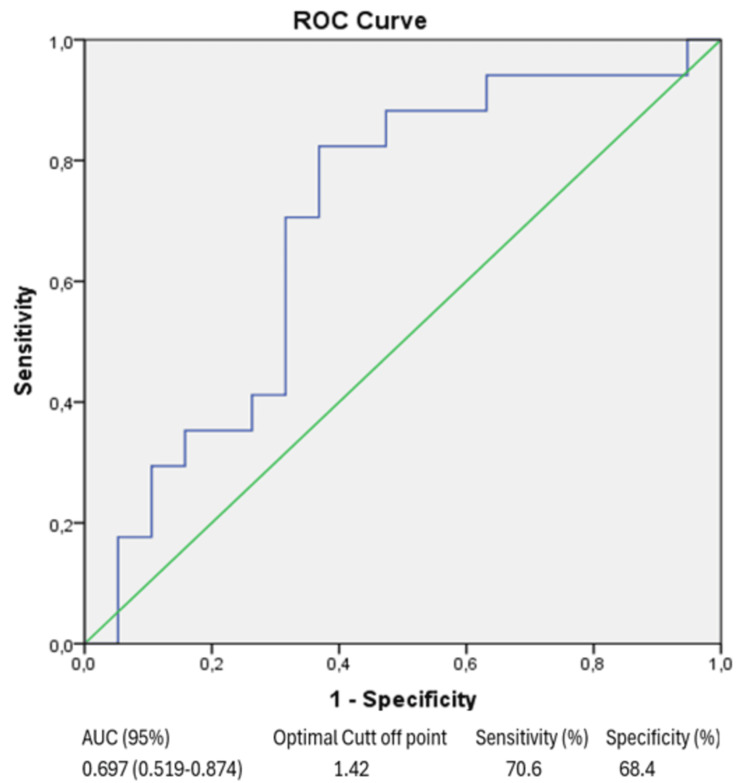
C-reactive protein/albumin ratio ROC analysis ROC: receiver operating characteristic; AUC: area under the ROC curve.

To evaluate the CAR value's utility in separating remission and active groups in elderly RA patients, ROC curve analysis was carried out. For the CAR value, ROC curve analysis yielded statistically significant results and the area under the ROC curve (AUC) was 0.697 (AUC 95% (confidence interval, CI) =0.697 (0.519-0.874), p < 0.044) (Figure 1). When the optimal cut-off value was accepted as ≥1.42, the sensitivity level was 70.6% and the specificity level was 68.4%.

## Discussion

The purpose of this study was to assess how CAR correlated with other clinical indicators in elderly individuals with RA. The DAS 28-ESR showed that patients in remission had lower CAR than those with active disease. Another noteworthy result was the significant correlation between CAR and CRP, ESR, and DAS28-ESR, but not with NLR. These findings may support the function of CAR in inflammatory diseases such as RA.

Similar to our findings, a study by Yang et al. [6] showed a positive association between CAR and CRP (r = 0.997, p < 0.001), ESR (p = 0.727, p < 0.001), and DAS 28-ESR (r = 0.645, p < 0.001). In another study similar to our study, Sunar et al. [7] found a weak relationship between DAS 28-ESR and CAR in RA patients (r = 0.327, p < 0.001). They stated that CAR may be an additional marker of disease activity in addition to other markers. Similar to our findings, the study by Fathy et al. [11] found that individuals with active RA had considerably greater CAR levels than those in remission (p < 0.001). In addition, Afifi et al. [12] and Elsabagh et al. [13] found a relation between DAS 28-ESR and CAR (r = 0.589, p = 0.001 and DAS28 low/moderate/high groups p = 0.024, respectively). On the other hand, several previous studies have shown that CAR is increased in many types of cancer, critically ill patients, vasculitis, Crohn's disease, and sepsis, which may suggest a relationship between inflammation and CAR [14-16]. It has also been reported that CAR is an independent predictor of mortality and performs better than CRP alone [6,17].

The accuracy of the CAR ratio in predicting RA activity was evaluated using ROC curve analysis. CAR had a specificity of 68.4%, an AUC of 0.697, a cut-off point of ≥1.42, and a sensitivity of 70.6% in our study. These results are comparable to those of Fathy et al. [11] who found that the accuracy of the CAR ratio in predicting RA activity in their study was 72.3% specificity and 79.1% sensitivity of CAR using ROC curve analysis. Afifi et al. [12] found that at a cut-off point of ≥1.66 (AUC: 0.789) and CAR had 66.6% specificity and 81.5% sensitivity. Elsabagh et al. [13] found that at a cut-off point of ≥2.66 (AUC 0.78), CAR had 81.3% sensitivity and 64.3% specificity.

To the best of our knowledge, this is the first study to look at the relationship between CAR and disease activity and quality of life in elderly patients with RA. In comparison to individuals in remission, we found that CAR was considerably greater in the group with high disease activity and CAR had a significant positive correlation with ESR, CRP, and DAS28-ESR. This finding could be seen as supporting the literature on the assessment of disease activity in elderly RA patients. However, our study found no discernible association between CAR and HAQ scores, which may be due to the low disease activity of the majority of patients. Similar to our study, no correlation was found between CAR and HAQ scores in recent studies [6,18].

The limitations of our research include the single center; the small sample size, which may weaken the generalization of the results; the lack of a follow-up assessment; and the lack of an evaluation of the correlation between CAR and other RA outcomes. Increasing the sample size in randomized controlled clinical trials will lead to more accurate results.

## Conclusions

The DAS 28-ESR is the most commonly used scale in clinics to assess disease activity in RA patients. However, the application of this scale in busy clinics is time-consuming. CAR is a readily calculable laboratory parameter that may help reduce the time needed to assess disease activity. The results of this study showed an association between CAR and disease activity in elderly RA patients. Our results suggest that CAR can be used as a cheap and easily applicable biomarker to determine inflammation and disease activity in clinics.
